# Characterizing the Contribution of Functional Microbiota Cultures in Pit Mud to the Metabolite Profiles of Fermented Grains

**DOI:** 10.3390/foods13111597

**Published:** 2024-05-21

**Authors:** Yingdong Wan, Jun Huang, Qiuxiang Tang, Suyi Zhang, Hui Qin, Yi Dong, Xiaojun Wang, Chuanfeng Qiu, Mengyang Huang, Zhu Zhang, Yi Zhang, Rongqing Zhou

**Affiliations:** 1 College of Biomass Science and Engineering, Sichuan University, Chengdu 610065, China; 2021223080043@stu.scu.edu.cn (Y.W.); junhuang@scu.edu.cn (J.H.); tangqiuxiang@scu.edu.cn (Q.T.); 2021223085074@stu.scu.edu.cn (Z.Z.); 2023223080027@stu.scu.edu.cn (Y.Z.); 2 Luzhou Lao Jiao Co., Ltd., Luzhou 646699, China; zhangsy@lzlj.com (S.Z.); qinhui@lzlj.com (H.Q.); dongyi1@lzlj.com (Y.D.); wangxj@lzlj.com (X.W.); qiucf@lzlj.com (C.Q.); huangmy@lzlj.com (M.H.)

**Keywords:** pit mud cultures, fermented grains, microbial community, flavor compounds

## Abstract

Elevating the flavor profile of strong flavors *Baijiu* has always been a focal point in the industry, and pit mud (PM) serves as a crucial flavor contributor in the fermentation process of the fermented grains (FG). This study investigated the influence of wheat flour and bran (MC and FC) as PM culture enrichment media on the microbiota and metabolites of FG, aiming to inform strategies for improving strong-flavor *Baijiu* flavor. Results showed that adding PM cultures to FG significantly altered its properties: FC enhanced starch degradation to 51.46% and elevated reducing sugar content to 1.60%, while MC increased acidity to 2.11 mmol/10 g. PM cultures also elevated FG’s ester content, with increases of 0.36 times for MC-FG_60d_ and 1.48 times for FC-FG_60d_ compared to controls, and ethyl hexanoate rising by 0.91 times and 1.39 times, respectively. Microbial analysis revealed that *Lactobacillus* constituted over 95% of the *Abundant bacteria* community, with *Kroppenstedtia* or *Bacillus* being predominant among *Rare bacteria*. *Abundant fungi* included *Rasamsonia*, *Pichia*, and *Thermomyces*, while *Rare fungi* consisted of *Rhizopus* and *Malassezia*. Metagenomic analysis revealed bacterial dominance, primarily consisting of *Lactobacillus* and *Acetilactobacillus* (98.80–99.40%), with metabolic function predictions highlighting genes related to metabolism, especially in MC-FG_60d_. Predictions from PICRUSt2 suggested control over starch, cellulose degradation, and the TCA cycle by fungal subgroups, while *Abundant fungi* and bacteria regulated ethanol and lactic acid production. This study highlights the importance of PM cultures in the fermentation process of FG, which is significant for brewing high-quality, strong-flavor *Baijiu*.

## 1. Introduction

Strong-flavor *Baijiu* was manufactured via distinctive solid-state fermentation craftwork, in which wheat and sorghum were used as primary raw materials, and participating microorganisms involved fungi, eubacteria, and archaea. The key microorganisms are sourced from *Daqu* and pit mud (PM). PM is a special type of clay lined at the bottom and four inner walls of the pit [1,2]. *Daqu* is an essential starter and one of the requisite raw materials in *Baijiu* brewing [3]. In fact, the contribution of the microbiota habituated in *Daqu* to the yield and quality of the fresh *Baijiu* still depended on the interaction with PM [4]. Previous studies revealed the synthesizing of some organic acids was driven by the synergistic effect of microbiota habiting in both FG and PM during the process [5]. The contribution of PM to the microbial community dynamics and microenvironmental parameters in FG reduced the abundance of dominant microbes, enriched functional microbial communities, and mitigated the acidification trend of the microenvironment [6]. Therefore, it is an effective strategy to improve the yield and quality of fresh *Baijiu* by regulating the interspecific interactions and their metabolic network among the microbiota, including within phases and between phases. In the past decade, results reported in many documents confirmed that bioturbation, or inoculating functional strains or microbiota, altered the endogenous microbiota structure and their metabolic dynamics in the brewing microecosystem, improving specific functional phenotypes. For example, adding 6% *Muqu* into *Qupei* (the initial non-fermentation starter) is an essential step in manufacturing high-quality *Jiangqu* [7]. In order to produce high-quality artificial pit mud (APM), the old PM was often used as the starter [8,9], and it not only accelerated the maturation rate of APM but also improved the content of ethyl hexanoate when the suspension of *Clostridium* spp. was inoculated [10]. The synthetic microbiota, composed of *Bacillus*, *Saccharomycopsis*, and *Absidia*, significantly improved the activity of amylase in *Daqu* [11].

Tetramethylpyrazine is one of the unique flavor components in *Baijiu*. The content of *Zaopei* and fresh *Baijiu* increased by 26 and 24 times, respectively, when the suspension of *B. amyloliquefaciens* was added in the initial stage of fermentation [12]. Similarly, the microbiota structure and their flavor metabolites in *Daqu* were improved by inoculating the synthetic microbiota, composed of *B. velezensis* and *B. subtilis* [13], which enhanced the relative abundance (RA) of some functional microorganisms, such as *Caproiciproducens*, *Clostridium*, *Methanobacterium*, *Methanosarcina*, etc., while decreasing that of *Lactobacillus* when it was used for *Baijiu* fermentation [14]. In addition, some research results have also shown that isolated strains from *Daqu* can effectively avoid defects in the production process. For example, two isolated strains of *Bacillus* high-degrading geosmin played a significant role in eliminating this odor [15]. *Kodamaea ohmeri* can effectively produce the biogenic amine [16], whereas *Lysinibacillus spherericus* can degrade ethyl carbamate and urea up to 41.77% and 63.32% in the *Baijiu* simulation system, respectively [17]. Meanwhile, the spatiotemporal characteristics of the *Zaopei* community are significant. With the progress of the fermentation process, the abundance of functional microbial groups, such as *Clostridium* and methanogenic archaea, gradually decreased, leading to a reduction in the yield and quality of the fresh *Baijiu* [18,19,20]. However, the role of cultures of PM functional microbiota on the fermentation of FG is still unclear.

This study investigated the effects of wheat flour and bran (MC and FC) served as the medium enriching functional microbiota in PM culture, respectively, on the physicochemical properties, volatile components, and microbial communities of FG simulated fermentation systems by the multi-omics method. Meanwhile, the contribution of rare and abundant microorganisms to the metabolite feature in FG was also examined. The objective is to establish a theoretical framework for the role of functional microbiota during FG fermentation and to facilitate the development of novel application technologies.

## 2. Materials and Methods

### 2.1. Preparation of High Ability of PM Culture Producing Ethyl Hexanoate

The PM originated from the pit with 100 years of Luzhou Laojiao Co., Ltd. (Luzhou city, China) and was domesticated repeatedly, as followed by a previous study [21]. The main process description is as follows: The PM was diluted 10 times with sterile normal saline and treated in an 85 °C hot water bath for 10 min to kill heat-labile microorganisms. Then, the suspension was inoculated into the modified acetate medium in a 5% ratio (*v*/*v*) and cultured at 37 ± 1 °C for 7 days. The total acid content of the cultures was quantified through alkaline titration. Concentrations of specific acids, namely acetic acid, butyric acid, and hexanoic acid, were analyzed using HPLC, which was composed of an Agilent 1260 HPLC system equipped with an Alltech OA-1000 organic acid column (300 mm × 7.8 mm, Agilent, Santa Clara, CA, USA). The samples underwent purification and filtration prior to analysis. Meanwhile, the quantities of colonies were measured using a hemocytometer. The sample with excellent results for these parameters was repeated according to the above process 10 times until these parameters were stabilized, referred to as functional microbiota (FM). FM was conserved at −20 °C for use as the initial starter, which was subcultured monthly to check its activity. The activated FM was inoculated into the sterile wheat flour and bran medium at a 5% ratio (*v*/*w*), respectively, and cultured at 37 ± 1 °C for 7 days. Subsequently, they were dried at 45 ± 2 °C and referred to as PM cultures, specifically MC and FC. PM cultures were placed in sterile self-sealing bags and stored at −20 °C for use in *Baijiu* brewing. All inoculation and sampling operations were conducted within a sterile workbench, ensuring a clean and independent cultivation environment to minimize the risks of contamination.

The formula for the modified sodium acetate medium was as follows: 5 g CH_3_COONa, 0.5 g (NH)_2_SO_4_, 0.4 g KH_2_PO_4_, 0.2 g Mg_2_SO_4_, 1.0 g yeast extract, 5.0 g CaCO_3_, and 1000 mL ddH_2_O, with the pH adjusted to 6.0–6.5. The medium was autoclaved at 121 °C for 20 min, followed by the addition of 20 mL of 100% ethanol [22]. The wheat flour or bran medium was prepared by mixing 1000 g of wheat flour or bran with 10.0 g of Na_2_CO_3_ and 1000 mL of distilled water. After thorough mixing, 150 g of the mixture was transferred into a 500 mL conical flask and autoclaved at 121 °C for 20 min.

### 2.2. Baijiu Brewing and Sampling

The experiment was carried out according to the process described in a previous study [23]. The initial FG, consisting of sorghum, *Huizao* (the last round of FG), and the pre-steamed rice husks, were combined in a ratio of 1:4:0.2. This mixture was steamed under atmospheric pressure for 1 h. After cooling to below 40 °C, *Daqu* and PM cultures were added to the initial FG based on the dry weight of sorghum, as detailed in Table 1. The mixture was transferred into simulated fermentation pits (6 L of plastic containers measuring 25 cm × 17 cm × 14 cm) and fermented at 30 °C for 60 days. Sampling occurred at 0 days, 30 days, and 60 days using the five-point method. After thorough mixing, the samples were divided into two portions, one stored at −20 °C for physicochemical and volatile substance analysis and the other stored at −80 °C for microbial community analysis.

### 2.3. Physicochemical Properties Analyzing

The moisture content of FG was measured by drying at 105 °C for 4 h and weighing. FG was mixed with CO_2_-free water at a ratio of 1:10 (*w*/*v*), extracted for 30 min, filtered, and then analyzed separately for acidity and reducing sugar content using acid-base titration and Fehling’s titration methods. The starch content was determined by hydrolyzing in a hydrochloric acid solution for 30 min, followed by using Fehling’s titration methods. Additionally, 100 g of FG were mixed with distilled water in a 1:2 ratio (*w*/*v*), and then 100 mL of distillate was obtained through distillation for alcohol content analysis, which was conducted using an alcoholometer. All the samples were measured in triplicate.

### 2.4. Detecting Volatiles

Volatile compounds were determined using headspace solid-phase microextraction gas chromatography–mass spectrometry (HS-SPME–GC–MS) on a Trace 1300-TSQ 9000 GC–MS system (Thermo Scientific, Waltham, MA, USA) equipped with a VF-WAX-MS capillary column (30.0 m × 0.25 mm × 0.25 µm, Agilent, Santa Clara, CA, USA).

Sample pretreatment [3]: 1.00 g of the sample and 10 μL of internal standard (methyl octanoate, 0.0073 g/100 mL) were added to a 20 mL headspace vial. The vial was placed on a constant-temperature magnetic stirrer at 60 °C for 15 min. Volatile components were extracted using a 50/30 µm DVB/CAR/PDMS fiber (2 cm, Supelco, Bellefonte, PA, USA) for 45 min. The extraction head was desorbed at 250 °C for 5 min. Chromatographic conditions: The injection port temperature was set at 250 °C, and the carrier gas was high-purity helium with a flow rate of 1 mL/min (>99.999%). The temperature program included an initial temperature of 40 °C for 5 min, followed by an increase at 4 °C/min to 100 °C, and then at 6 °C/min to 230 °C, where it was held for 10 min. Mass spectrometry conditions: The ion source temperature was 250 °C; the transfer line temperature was 300 °C; and the ionization was performed by electron impact (EI) at 70 eV. The scanning range was 35 to 400 amu. Qualitative and quantitative analysis: After comparing the obtained mass spectra with the NIST 2017 mass spectral library, only compounds with a similarity greater than 80% were retained for further analysis. The content of each volatile compound was calculated based on the ratio of its peak area to the content of the internal standard, methyl octanoate. All the samples were measured in triplicate.

### 2.5. Amplicon Sequencing

Total DNA was extracted from the samples using the Fast DNA SPIN extraction kit (MP Biomedicals, Santa Ana, CA, USA) following the manufacturer’s instructions. The quality of DNA extraction was verified using 0.8% agarose gel electrophoresis and quantified using a spectrophotometer (Thermo Scientific, Waltham, MA, USA). The bacterial 16S rRNA V3-V4 region and the fungal ITS1 region were amplified using universal primers 338F/806R and ITS5/ITS1, respectively.

Polymerase chain reaction (PCR) system: The PCR reaction mixture included 5 µL of 5× Q5 high-fidelity reaction buffer, 5 µL of 5× Q5 high-fidelity GC buffer, 0.25 µL of Q5 high-fidelity DNA polymerase (5 U/µL), 2 µL of dNTPs (2.5 mmol/L), 1 µL each of forward and reverse primers (10 µmol/L), 2 µL of DNA template, and 8.75 µL of ddH_2_O. The amplification program consisted of an initial denaturation at 98 °C for 2 min, followed by 25 cycles of denaturation at 98 °C for 15 s, annealing at 55 °C for 30 s, extension at 72 °C for 30 s, and a final extension at 72 °C for 5 min. PCR products were purified using VAHTSTM DNA Clean Beads (Vazyme, Nanjing, China), and the concentration was quantified using the Quant-iT PicoGreen dsDNA Assay Kit (Invitrogen, Carlsbad, CA, USA). After purification, samples were sequenced on the Illumina Novaseq platform by Shanghai Personal Biotechnology Co., Ltd. (Shanghai, China).

The raw sequencing data were primarily processed using QIIME2. The demux plugin was used for multiplexing, and cutadapt was employed to remove primers. Subsequently, DADA2 was applied for quality filtering, denoising, merging, and removing chimeric sequences. Further filtering retained amplicon sequence variants (ASVs) found in only one sample and singleton sequences. Finally, the taxonomic assignment was performed against the Silva (v. 132) and UNITE (v. 8.0) databases.

### 2.6. Metagenomic Sequencing

Total microbial DNA extraction was performed using the Mag-Bind Soil DNA Kit (Omega Bio-Tek, Norcross, GA, USA). The extracted DNA was quantified using a Qubit 4 fluorometer (Thermo Scientific, Waltham, MA, USA), and the quality was assessed by agarose gel electrophoresis. Library construction followed the Illumina TruSeq DNA library preparation protocol, including DNA fragmentation, repair, introduction of “A” bases, adapter ligation, purification, and enrichment of DNA fragments. The library was validated using Qubit 4 and Agilent 2100 after normalization and subjected to Illumina sequencing.

### 2.7. Statistical Analysis

Visualizations of physicochemical properties were conducted using OriginPro 2019b (Origin Lab Corporation, MA, USA). The differences between the statistical significance (*p* < 0.05) and the means of the samples were tested by one-way analysis of variance (ANOVA) using SPSS 25.0 software (SPSS Inc., Chicago, IL, USA). Specifically, data normality was assessed with P-P and Q-Q plots and the Shapiro–Wilk test. Variance homogeneity was tested, followed by either the F-test for homogeneous or the Welch test for heterogeneous variances to compare group means. Post hoc comparisons used Duncan, Tukey, or Tamhane T2 tests. The microbial community assessment included α-diversity metrics such as the Chao1 index and Shannon index, as well as β-diversity metrics using principal component analysis (PCA) and hierarchical clustering analysis. Metabolic functions were predicted based on the KEGG, Metacyc, and CAZy databases using PICRUSt2. Partial least squares discriminant analysis (PLS-DA) and permutation tests were performed in SIMCA 14.1. Heatmaps were generated using TB Tools (v. 1.108).

## 3. Results and Discussion

### 3.1. Changes in Physicochemical Properties of FG

The physicochemical properties of FG exhibited distinct trends throughout the whole process, as documented in Table 2. During the initial phase (0–30 days), the moisture content increased significantly and then stabilized in the later phase (30–60 days), up by about 11%, in line with past research [18,24]. This consistent trend across all samples suggested normal microbial metabolism in each group [24]. Microorganisms initiated fermentation, hydrolyzing starch to reducing sugars that were then converted into alcohols, acids, and flavor components, with starch and reducing sugar consumption reflecting the process [20,24]. The starch content gradually decreased throughout the process, especially in the early phase [18]. Notably, on day 30, the starch degradation rates of DZ-FG, MC-FG, and FC-FG were 40.43%, 32.61%, and 33.64%, respectively. By the end of fermentation, these rates had increased to 43.88%, 36.76%, and 51.46%, with FC-FG exhibiting significant further degradation in the later phase. Acidity showed an overall increasing trend, with more pronounced increases observed in the later phase. Notably, the acidity of MC-FG was significantly higher than that of DZ-FG and FC-FG, both on day 30 and day 60. The trends in reducing sugar content varied markedly among samples. DZ-FG showed a notable decrease in the early phase, followed by a slight rise in the later phase. Conversely, MC-FG increased in the early phase, followed by a significant decline in a later phase, possibly due to the conversion of reducing sugars to acids, as indicated by its significantly higher acidity. FC-FG’s reducing sugar content decreased slightly in the early phase and then increased significantly in a later phase, which corresponded with its substantial starch degradation in the later phase, while its acidity remained similar to that of DZ-FG. The alcohol content increased significantly in the early phase, peaking in DZ-FG_30d_, aligning with its highest rates of starch and reducing sugar degradation [20]. Subsequently, the alcohol content slightly decreased in a later phase, possibly due to its conversion into esters. The changes in physicochemical properties during the simulated fermentation closely mirrored those observed in industrial-scale *Baijiu* fermentation, underscoring the effectiveness of the simulation in replicating real-world fermentation dynamics [18,20].

### 3.2. Changes in Volatile Components

**Figure 1:**
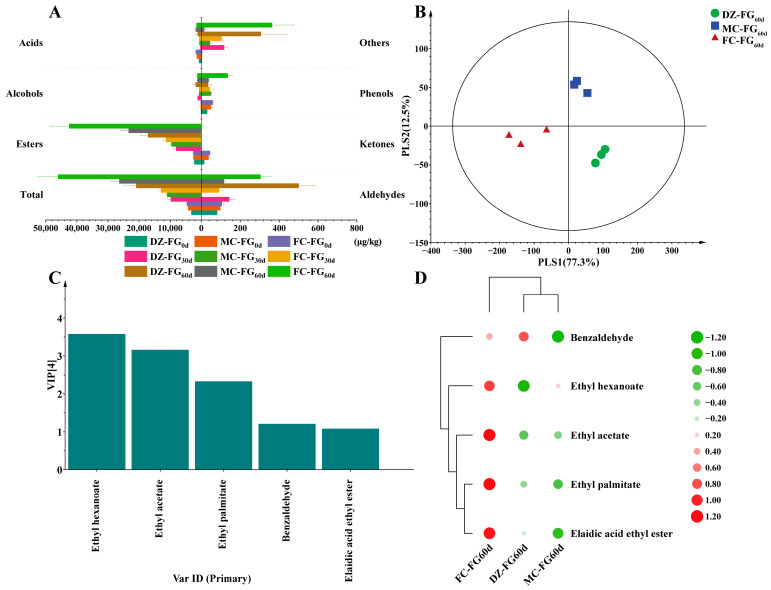
(**A**) Difference in the content of volatile components of FG; (**B**) PLS-DA of volatile components of FG_60d_; (**C**) five distinct volatiles were identified as crucial by VIP > 1; (**D**) displaying the differences of these five volatile compounds through heatmap.

In the present study, 68 volatiles identified in these FG were divided into seven groups: esters (36), alcohols (6), acids (8), aldehydes (7), ketones (2), phenols (5), and others (4). The contents of volatiles increased from 3.14 mg/kg–4.61 mg/kg to 9.79–12.97 mg/kg when fermented for 30 days, reaching 20.92–46.02 mg/kg at the end. Subsequently, the increase ranged from 5.34 times (MC-FG) to 8.98 times (FC-FG) by the end of fermentation (Figure 1A). Among these volatiles, esters, alcohols, and acids were predominant, echoing findings reported by previous studies [24,25]. The contents of esters rose by 3.67 times–4.47 times and 2.11 times–3.73 times in the prophase and the anaphase of fermentation, respectively. In comparison to DZ-FG_60d_’s, the ester content in MC-FG_60d_ and FC-FG_60d_ increased by 0.36 times and 1.48 times, respectively. Notably, ethyl hexanoate, contributing to fruity aromas for *Baijiu* [23], had the highest content, increasing by 0.91 times and 1.39 times in MC-FG_60d_ and FC-FG_60d_, respectively, compared to DZ-FG_60d_. Additionally, at the end of fermentation, the volatile acid content also increased due to the addition of MC and FC, with an increase of 0.51 times and 0.30 times in MC-FG_60d_ and FC-FG_60d_, respectively, compared to DZ-FG_60d_. The elevated volatile acid content of MC-FG_60d_, consistent with its significantly higher acidity, may have suppressed the activity of its functional microbial community [26], leading to less pronounced flavor enhancement relative to FC-FG_60d_. However, by the end of fermentation, the alcohol content in MC-FG and FC-FG decreased to 0.67 times and 0.70 times that of DZ-FG, recording contents of 1.20 mg/kg and 1.26 mg/kg, respectively. The decrease was mainly attributed to the reduction in branched-chain alcohols such as phenethyl alcohol, 2,3-butanediol, and isoamyl alcohol, which was consistent with the previous study on the impact of PM on fermentation flavor [2]. These observations suggested that FC notably enhanced the content of volatiles in FG, especially esters and ethyl hexanoate.

The differentiation of characteristic volatiles in FG was effectively determined using a partial least squares discriminant analysis (PLS-DA) model (Figure 1B). The model demonstrated outstanding performance with values of R2X (cumulative) at 0.992, R2Y (cumulative) at 0.988, and Q2 (cumulative) at 0.958, indicating exceptional explanatory power for both the X and Y matrices and a strong predictive capability [23,27]. The analysis revealed a significant distinction in volatile profiles among the different types of FG, with DZ-FG_60d_, MC-FG_60d_, and FC-FG_60d_ positioned in the IV, I, and III quadrants, respectively, highlighting the marked differences in their volatile compositions.

Based on the variable importance of projection scores (VIP > 1.0), five distinct volatiles were identified as crucial [27,28], namely ethyl hexanoate, ethyl acetate, ethyl palmitate, benzaldehyde, and elaidic acid ethyl ester (Figure 1C). Ethyl hexanoate and ethyl acetate, key structural components, significantly influence the quality of fresh *Baijiu* through their concentration and the ratio between them [29,30]. Benzaldehyde, in moderate amounts, enhances the taste, whereas excessive amounts can impart a bitter flavor [31]. The cluster heatmap of characteristic volatiles indicated that benzaldehyde had the highest concentration in DZ-FG_60d_, while the concentrations of the other four volatiles were significantly higher in FC-FG_60d_ (Figure 1D).

### 3.3. Difference in Microbiota Diversity and Their Constructure among Samples

The differences in α-diversity of the microbial communities are presented in Table 3. The bacterial richness and diversity in DQ were higher than those in MC and FC, possibly due to the greater ease of colonization by bacteria in smaller habitats [32,33]. The fungal diversity in MC was higher than that in FC and DQ, with the latter exhibiting the lowest diversity. In the initial phase, bacterial richness was highest in FC-FG_0d_, while diversity was highest in MC-FG_0d_. For fungi, richness and diversity were both highest in FC-FG_0d_. After 60 days of fermentation, there was a significant reduction in bacterial richness and diversity, along with a decrease in fungal richness, consistent with the findings reported by a previous study [34].

**Figure 2:**
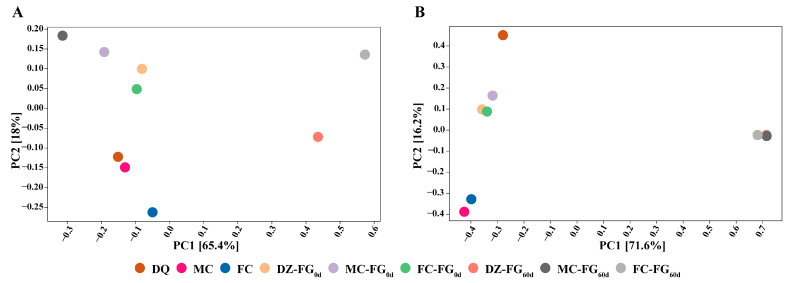
PCA analysis of microbial communities. (**A**) Fungi; (**B**) bacteria.

The results of the fungal community PCA analysis are presented in Figure 2A. For the fungal community, at the initial stage (0 days), the distances between FG were relatively short, indicating a similarity in structure. However, after 60 days, while the structure of MC-FG_60d_ remained similar to the initial state, FC-FG_60d_ and DZ-FG_60d_ were not only distanced from the initial state but were also moved away from MC-FG_60d_. At the initial stage, the structure of the bacterial community resembled that of fungi, with mutual structural similarity, and a higher degree of similarity between FC-FG_0d_ and DZ-FG_0d_ was observed. Before and after fermentation, the bacterial community structures between the FG were found to be in a dispersed state, but aggregated states were individually demonstrated (Figure 2B). These results revealed that a significant impact on the structure of the fungal community was exerted by FC.

### 3.4. Distribution of Abundant and Rare Microbial Taxa

The samples were divided into three groups: *Daqu* and PM cultures (DQ, MC, and FC), FG_0d_ (DZ-FG_0d_, MC-FG_0d_, and FC-FG_0d_), and FG_60d_ (DZ-FG_60d_, MC-FG_60d_, and FC-FG_60d_). In reference to the definition provided by the previous study [35], the samples within the same group were categorized into the following six classes: Always abundant taxa (AAT): Within the same group, the relative abundance of ASVs was ≥1% in all samples. Conditionally abundant taxa (CAT): Within the same group, the relative abundance of ASVs was ≥1% in some samples but never rare (<0.01%) in any samples. Always rare taxa (ART): Within the same group, the relative abundance of ASVs was <0.01% in all samples. Conditionally rare taxa (CAT): Within the same group, the relative abundance of ASVs was <0.01% in some samples but never abundant (≥1%) in any samples. Moderate taxa (MT): Within the same group, the relative abundance of ASVs was between 0.01% and 1% in all samples. Conditionally rare and abundant taxa (CRAT): Within the same group, the relative abundance of ASV ranged from rare (<0.01%) to abundant (≥1%). AAT, CAT, and CRAT were classified as abundant taxa, while ART and CRT were categorized as rare taxa [36].

The distribution of abundant and rare taxa in the microbiota revealed that 10.92% of bacterial ASVs and 14.81% of fungal ASVs were classified as abundant taxa (AT), accounting for 94.39% and 98.37% of the total sequence counts, respectively, in *Daqu* and PM cultures (Table 4). Conversely, 86.55% of bacterial ASV and 77.78% of fungal ASV were identified as rare taxa (RT), comprising only 5.17% and 1.14% of the total sequence counts, respectively. This pattern of a lower proportion of ASVs but a higher proportion of sequence counts for AT and opposition for RT was consistent across both the initial and final FG, indicating a similar microbial composition trend [37].

**Figure 3:**
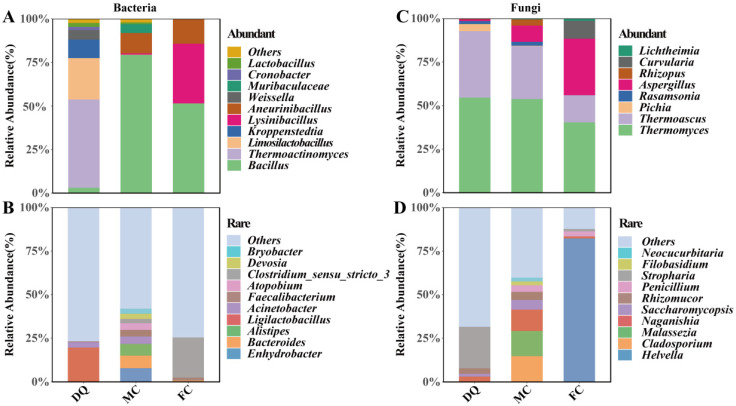
Abundant and rare microbial composition of *Daqu* and PM cultures. (**A**) *Abundant bacteria*; (**B**) *Rare bacteria*; (**C**) *Abundant fungi*; (**D**) *Rare fungi*.

In *Daqu*, as well as two types of PM cultures, 13 genera of AT bacteria were identified, including *Thermoactinomyces*, *Limosilactobacillus*, *Kroppenstedtia*, *Weissella*, etc., which dominated in DQ, while *Bacillus* and *Aneurinibacillus* shared in both MC and FC, and *Lysinibacillus* also dominated in the latter (Figure 3A). *Kroppenstedtia*, known for its high amylase and lipase secretion and organic acid metabolism [38], along with *Bacillus*, was highlighted as a functional bacteria in *Daqu* [39]. *Aneurinibacillus*, notable for its cellulose degradation of various enzymes, such as alkyl or aryl transferases, methyl transferases, and secretion abilities, was first detected in PM cultures, contributing to the formation of methyl esters [40]. Therefore, its role in the *Baijiu* brewing process warrants further exploration. *Lysinibacillus* showed the potential to efficiently degrade ethyl carbamate and its precursor, urea [17]. *Limosilactobacillus* was predominant in the early *Daqu* manufacturing process, converting substrates into lactic acid and ethanol [41]. The bacterial RT comprised 103 genera, with the dominated *Ligilactobacillus*, *Acinetobacter*, etc., in DQ, and *Enhydrobacter*, *Bacteroides*, *Alistipes*, etc., dominated in MC, as well as *Clostridium_sensu_stricto_3* in FC (Figure 3B).

These starters contained eight genera of *Abundant fungi*, with *Thermomyces* and *Thermoascus* being common in both DQ and MC, and the order of RA was decreased in sequence (Figure 3C). The third most *Abundant fungus* was *Pichia* in DQ and *Aspergillus* in MC, respectively. *Curvularia* was uniquely abundant in FC, indicating a variation in dominant fungal genera across starters. In both *Thermomyces* and *Thermoascus*, known for their temperature tolerance and hydrolase production, the former produced temperature tolerance cellulases and proteases, while the latter produced hydrogen peroxide, endoglucanases, and glucosidases. Therefore, it can increase the yield and quality of fresh *Baijiu* by enhancing the ability to degrade macromolecular substrates, as well as retarding the toxicity of hydrogen peroxide to cells when the RA of these dominant microorganisms increases [42,43]. The fungal RT consisted of 42 genera, with the highest RA *Stropharia* in DQ and the most dominant *Cladosporium*, *Malassezia*, and *Naganishia* in MC, as well as the overwhelmingly dominant *Helvella* in FC (Figure 3D).

**Figure 4:**
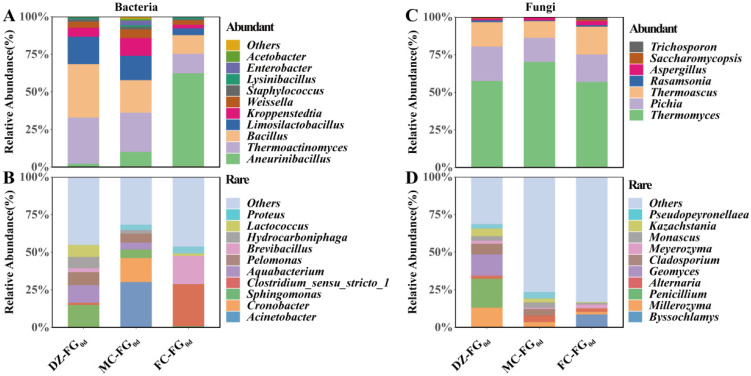
Abundant and rare microbial composition of FG_0d_. (**A**) *Abundant bacteria*; (**B**) *Rare bacteria*; (**C**) *Abundant fungi*; (**D**) *Rare fungi*.

There were 11 genera classified as AT bacterial microbiota in the initial FG, but the RA of these microorganisms was different among samples, although their composition was similar. In each of DZ-FG_0d_ and MC-FG_0d_, the RA of *Thermoactinomyces*, *Bacillus*, and *Limosilactobacillus* was higher, while that of *Aneurinibacillus* exceeded 60% in FG-FG_0d_ (Figure 4A). The RT bacterial microbiota comprised 65 genera. The species and genus with higher RA were completely different among samples, in which *Sphingomonas*, *Aquabacterium*, and *Pelomonas*, as well as *Acinetobacter* and *Cronobacter*, were higher in DZ-FG_0d_ and MC-FG_0d_, respectively, but *Brevibacillus* and *Clostridium_sensu_stricto_1* dominated in FC-FG_0d_ (Figure 4B).

The fungal AT comprised 7 genera, including *Thermomyces*, *Pichia*, *Thermoascus*, etc. (Figure 4C), while the RT of the fungal microbiota involved 35 genera, in which *Millerozyma*, *Penicillium*, and *Geomyce* dominated in DZ-FG_0d_ and unidentified fungi were predominant in both MC-FG_0d_ and FC-FG_0d_ (Figure 4D).

**Figure 5:**
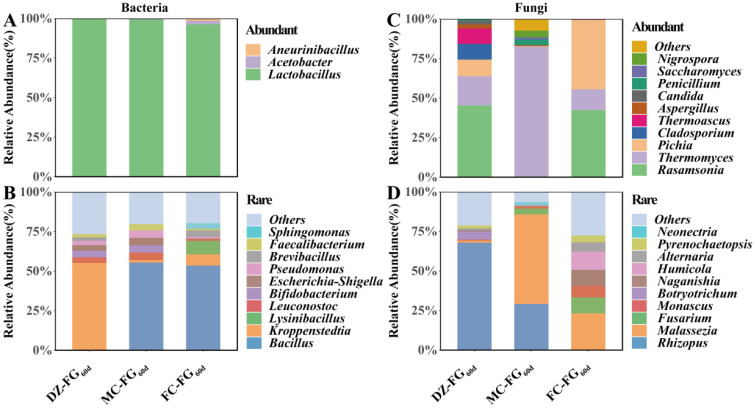
Abundant and rare microbial composition of FG_60d_. (**A**) *Abundant bacteria*; (**B**) *Rare bacteria*; (**C**) *Abundant fungi*; (**D**) *Rare fungi*.

By the time fermentation finished, the bacterial microbiota had belonged to AT and comprised three genera: *Lactobacillus*, with RA > 95%, which agreed with the result reported by the previous study [44], dominated, followed by *Aneurinibacillus* (Figure 5A). The bacterial microbiota belonging to RT involved 41 genera: *Kroppenstedtia* and *Bacillus* dominated in DZ-FG_60d_ and both MC-FG_60d_ and FC-FG_60d_, respectively (Figure 5B).

The AT fungal microbiota consisted of 11 genera: *Rasamsonia* and *Cladosporium* were transformed into AT in DZ-FG_60d_, along with *Thermomyces*, *Pichia*, and *Thermoascus*, which were also AT in the initial phase (Figure 5C). However, RA of *Thermomyces* accounted for 82.38% in MC-FG_60d_, while *Rasamsonia*, *Thermomyces*, and *Pichia* dominated in FC-FG_60d_. *Rasamsonia* is shared in both DZ-FG_60d_ and FC-FG_60d_ and is often detected in FG of strong-flavor *Baijiu* [26], and the contribution characterization needs to be explored further. RT of the fungal microbiota comprised 27 genera, of which *Millerozyma* evolved into *Rhizopus* in DZ-FG_60d_ and enhanced RA of *Malassezia* significantly in MC-FG_60d_, as were that of *Malassezia*, *Fusarium*, *Humicola*, *Naganishia*, etc., in FC-FG_60d_ (Figure 5D). Among these species and genera classified as RT fungal microbiota, *Malassezia* and *Fusarium* were major functional fungi [37], which might improve the quality of the base liquor as their RA enhanced.

### 3.5. Differences in Microbiota Composition and Functional Annotation among FG by Metagenomic Sequencing

**Figure 6:**
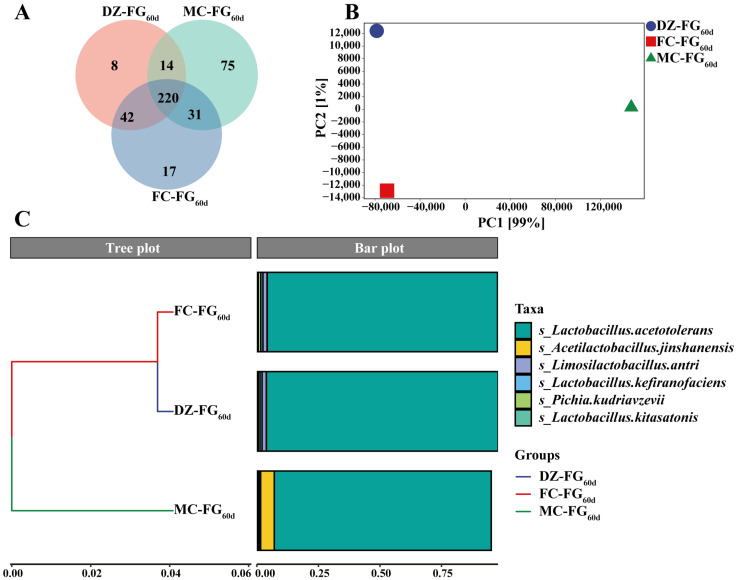
Differences in the microbial community of FG_60d_ at the species level. (**A**) Based on the Venn diagram, shared and unique species in FG_60d_ were demonstrated. The degree of differences between microbial communities in FG_60d_ was analyzed through (**B**) PCA and (**C**) hierarchical cluster analysis.

As shown in Figure 6A, a total of 220 shared species were detected in FG_60d_, of which 8, 17, and 75 unique species were found in DZ-FG_60d_, FC-FG_60d_, and MC-FG_60d_, respectively. Among these species identified, the bacteria dominated, primarily consisting of *Lactobacillus* and *Acetilactobacillus*, which accounted for 99.02%, 99.4%, and 98.80% of the total microbial population in DZ-FG_60d_, FC-FG_60d_, and MC-FG_60d_, respectively. These findings are in agreement with the results obtained by amplicon sequencing (Table 5). Specifically, *Lactobacillus acetotolerans* and *Acetilactobacillus jinshanensis* were dominant, with their combined RA reaching 97.38%, 99.26%, and 97.14%, respectively, in DZ-FG_60d_, FC-FG_60d_, and MC-FG_60d_. The total RA of the fungal microbiota varied from 0.06% to 0.55%, mainly comprised of *Pichia* and *Saccharomyces*, consistent with the result of amplicon sequencing. However, *Rasamsonia* and *Thermomyces* were undetected as the RA was too low in the overall analysis of bacterial and fungal microbiota.

The PCA results are displayed in Figure 6B. PC1 and PC2 accounted for 99% and 1% of the variance in species community composition, respectively. The projection on PC1 showed that DZ-FG_60d_ and FC-FG_60d_ were very close to each other, indicating similarity in their species communities, whereas MC-FG_60d_ was distinctly separated from these two, highlighting a significant difference in its microbiota structure. Similarly, the PCA findings were corroborated by the results from hierarchical cluster analysis (Figure 6C). These results revealed that the microbiota structures of DZ-FG_60d_ and FC-FG_60d_ were similar to each other but significantly different from those of MC-FG_60d_.

**Figure 7:**
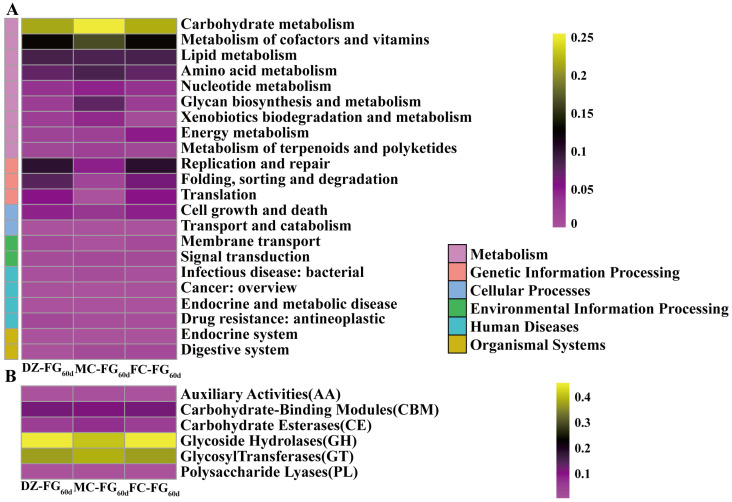
Distribution of KEGG and CAZy genes in different categories of FG_60d_. (**A**) KEGG annotation; (**B**) CAZy annotation.

Based on the KEGG annotation, differences in the metabolic functional profile among FG were inferred (Figure 7A). The RA of genes related to metabolism was highest, primarily enriched in carbohydrate metabolism [45], with the highest value of 87.14% observed in MC-FG_60d_. This was followed by genetic information processing, which had a higher RA in both DZ-FG_60d_ and FC-FG_60d_. Conversely, the RA of genes related to human diseases was lower in both FC-FG_60d_ and MC-FG_60d_.

Utilizing CAZy annotation, the composition of the enzymes related to carbohydrate metabolism was investigated (Figure 7B). These results revealed that the RA of expressed glycoside hydrolases (GH) and glycosyl transferases (GT) was the highest [44], and carbohydrate-binding modules (CBM) and carbohydrate esterases (CE) also showed relatively high abundance. GH enzymes catalyze the hydrolysis of glycosidic bonds to break down complex sugars and polysaccharides [46]. GT enzymes played a crucial role in polysaccharide synthesis or glycosylation processes [47] and were instrumental in the Embden–Meyerhof–Parnas (EMP) pathway and the hexose monophosphate (HMP) pathway, facilitating the use of glucose for lactic acid production [44]. In MC-FG_60d_, a lower RA of GH was observed, whereas GT exhibited a higher RA, correlating with its increased starch content and reduced sugar content.

### 3.6. Differences in the Contribution of Abundant and Rare Taxa to Metabolic Pathways

**Figure 8:**
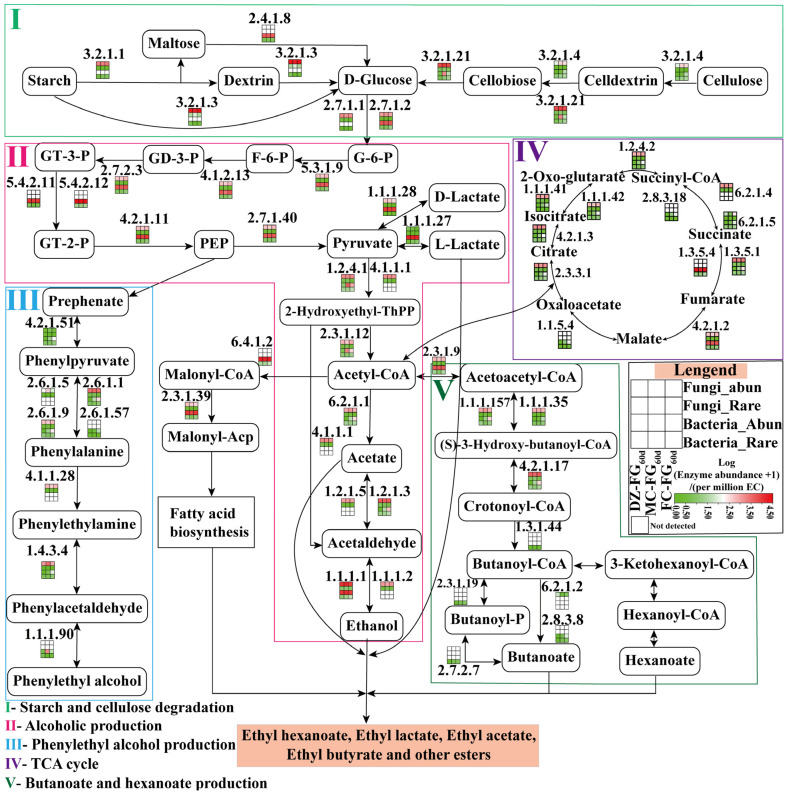
Functional diversity of abundant and *Rare bacterial* and fungal subcommunities and their contribution to major metabolic pathways of FG_60d_ based on PICRUSt2 prediction.

The predictive results from PICRUSt2 indicated that the metabolic pathways associated with flavor synthesis were explored, including the degradation of starch and cellulose, ethanol production, phenylethanol production, the TCA cycle, and pathways for butyric and hexanoic acid generation (Figure 8). Starch and cellulose were broken down into glucose through the action of enzymes such as alpha-amylase (EC:3.2.1.1), glucoamylase (EC:3.2.1.3), maltose phosphorylase (EC:2.4.1.8), cellulase (EC:3.2.1.4), and beta-glucosidase (EC:3.2.1.21). Among these enzymes, maltose phosphorylase (EC:2.4.1.8) was mainly regulated by the abundant bacterial subgroups, while the others were regulated by the abundant fungal subgroups.

Glucose was converted into pyruvic acid, a key precursor for various flavor compounds, through glycolysis [48]. In aerobic conditions, pyruvic acid was transformed into acetyl-CoA, which was essential for the tricarboxylic acid (TCA) cycle and fatty acid synthesis. Under anaerobic conditions, pyruvic acid could be catalyzed by lactate dehydrogenase to produce lactic acid or undergo decarboxylation to generate acetaldehyde, which is subsequently reduced to ethanol. Lactic acid production involved both *Abundant fungi* and bacteria, with the L-lactic acid-producing enzyme (EC:1.1.1.27) regulated by *Abundant bacteria* and the D-lactic acid-producing enzyme (EC:1.1.1.28) regulated by both. Pyruvate decarboxylase (EC:4.1.1.1) was mainly regulated by *Abundant fungi*, while the crucial enzymes (EC:1.1.1.1 and EC:1.1.1.2) for ethanol production were co-regulated by both *Abundant bacteria* and fungi.

The metabolism of phenylalanine was primarily regulated by the fungal community, although the crucial enzyme (EC:1.1.1.90) for the synthesis of phenylethanol was identified exclusively in the bacterial microbiota. The TCA cycle, essential for aerobic respiration, plays a vital role in providing energy and nutrition for microbial growth and is primarily influenced by *Abundant fungi*. Meanwhile, the metabolism of the butyric acid pathway was strongly associated with the bacterial RT [4].

## 4. Conclusions

This study employed HS-SPME–GC–MS and high-throughput sequencing to systematically investigate the impact of PM cultures on the volatile components, microbial community structure, and metabolic profiles of FG. Results indicated that the addition of MC and FC enhanced the flavor of FG, notably increasing the content of esters and ethyl hexanoate. FC culture exerted a more pronounced effect, enriching key flavor components such as ethyl hexanoate, ethyl acetate, ethyl palmitate, and elaidic acid ethyl ester. PM cultures induced notable shifts in the composition of rare taxa: dominant *Rare bacteria* transitioned from *Kroppenstedtia* to *Bacillus*, and dominant *Rare fungi* shifted from *Rhizopus* to *Malassezia*, *Fusarium*, *Humicola*, and *Naganishia*. Through the synergistic effects of abundant and rare taxa, their metabolic functional profiles were enhanced, leading to the accumulation of flavor substances.

In conclusion, this study provides essential insights into the role of PM functional microbial cultures in influencing the flavor and microbial community dynamics of FG. These findings are instrumental in advancing the regulation of the production processes, resulting in the creation of high-quality *Baijiu*. This research underscores the importance of microbial diversity and interactions within PM cultures for optimizing and innovating *Baijiu* production.

## Figures and Tables

**Table 1:** Details and abbreviation of the samples.

Sample	The Date of Samples	The Ratios of *Daqu* or PM Cultures (*w*/*w*)		
		*Daqu* (DQ)	MC	FC
DZ-FG_0d_	The 0 days of DZ-FG	15%	-	-
MC-FG_0d_	The 0 days of MC-FG	15%	1%	-
FC-FG_0d_	The 0 days of FC-FG	15%	-	1%
DZ-FG_30d_	The 30th day of DZ-FG	15%	-	-
MC-FG_30d_	The 30th day of MC-FG	15%	1%	-
FC-FG_30d_	The 30th day of FC-FG	15%	-	1%
DZ-FG_60d_	The 60th day of DZ-FG	15%	-	-
MC-FG_60d_	The 60th day of MC-FG	15%	1%	-
FC-FG_60d_	The 60th day of FC-FG	15%	-	1%

“-“ indicates that it was not added.

**Table 2:** Differences in physicochemical properties of FG.

Sample	Moisture (%)	Starch (g/100 g)	Reducing Sugar (g/100 g)	Acidity (mmol/10 g)	Alcohol (%)
DZ-FG_0d_	57.96 ± 0.26	23.45 ± 0.24 ^a^	1.55 ± 0.06 ^a^	0.85 ± 0.00 ^b^	ND
MC-FG_0d_	57.53 ± 0.32	22.66 ± 0.22 ^b^	1.33 ± 0.03 ^b^	0.92 ± 0.04 ^ab^	ND
FC-FG_0d_	57.33 ± 0.00	23.90 ± 0.25 ^a^	1.39 ± 0.09 ^b^	0.95 ± 0.03 ^a^	ND
DZ-FG_30d_	64.61 ± 0.14	13.97 ± 0.45 ^b^	1.15 ± 0.02 ^c^	1.00 ± 0.01 ^b^	8.43 ± 0.45 ^a^
MC-FG_30d_	63.55 ± 0.23	15.27 ± 0.05 ^a^	1.37 ± 0.06 ^a^	1.09 ± 0.02 ^a^	7.20 ± 0.50 ^b^
FC-FG_30d_	64.22 ± 0.57	15.86 ± 0.16 ^a^	1.25 ± 0.00 ^b^	1.00 ± 0.01 ^b^	7.70 ± 0.50 ^ab^
DZ-FG_60d_	65.91 ± 0.53	13.16 ± 0.50 ^a^	1.18 ± 0.03 ^b^	1.88 ± 0.01 ^b^	7.00 ± 0.20
MC-FG_60d_	64.52 ± 0.90	14.33 ± 0.26 ^a^	0.84 ± 0.00 ^c^	2.11 ± 0.03 ^a^	6.47 ± 0.15
FC-FG_60d_	63.92 ± 0.34	11.60 ± 1.14 ^b^	1.60 ± 0.01 ^a^	1.83 ± 0.01 ^b^	6.70 ± 0.50

Data are presented as means ± standard deviations (*n* = 3). “ND” indicates that it was not detected in the sample. Values with different letters within a row are significantly different statistically (*p* < 0.05).

**Table 3:** α-diversity index difference.

Sample	Bacteria				Fungi			
	Chao1	Observed Species	Shannon	Simpson	Chao1	Observed Species	Shannon	Simpson
DQ	234.06	227.60	4.08	0.84	69.49	68.50	1.85	0.60
MC	216.87	215.00	3.58	0.77	95.76	94.40	2.44	0.66
FC	141.16	141.00	3.59	0.87	44.80	44.30	2.38	0.75
DZ-FG_0d_	131.23	130.50	4.60	0.92	63.17	62.10	2.24	0.64
MC-FG_0d_	143.97	142.80	4.78	0.93	65.51	63.20	1.84	0.50
FC-FG_0d_	181.07	161.30	3.41	0.74	71.67	69.60	2.44	0.66
DZ-FG_60d_	52.39	46.20	1.44	0.55	49.14	45.90	2.50	0.74
MC-FG_60d_	40.24	39.80	1.84	0.62	54.23	52.60	1.81	0.46
FC-FG_60d_	47.07	45.70	1.57	0.51	37.03	37.00	2.13	0.68

**Table 4:** Distribution of abundant and rare taxa.

		Classification	AAT	CAT	CRAT	ART	CRT	MT	Abundant Taxa	Rare Taxa
Bacteria	*Daqu* and PM cultures	ASV proportion	0.84%	0.84%	9.24%	18.49%	68.07%	2.52%	10.92%	86.55%
		Relative abundance	41.31%	1.04%	52.04%	0.05%	5.11%	0.44%	94.39%	5.17%
	FG_0d_	ASV proportion	7.29%	3.13%	1.04%	23.96%	43.75%	20.83%	11.46%	67.71%
		Relative abundance	91.03%	2.25%	0.96%	0.03%	1.35%	4.38%	94.24%	1.38%
	FG_60d_	ASV proportion	2.22%	0.00%	4.44%	60.00%	31.11%	2.22%	6.67%	91.11%
		Relative abundance	97.88%	0.00%	1.43%	0.06%	0.30%	0.34%	99.31%	0.36%
Fungi	*Daqu* and PM cultures	ASV proportion	3.70%	5.56%	5.56%	33.33%	44.44%	7.41%	14.81%	77.78%
		Relative abundance	76.29%	16.78%	5.30%	0.04%	1.10%	0.49%	98.37%	1.14%
	FG_0d_	ASV proportion	8.70%	6.52%	0.00%	52.17%	23.91%	8.70%	15.22%	76.09%
		Relative abundance	96.92%	2.27%	0.00%	0.05%	0.27%	0.49%	99.19%	0.32%
	FG_60d_	ASV proportion	2.63%	18.42%	7.89%	50.00%	21.05%	0.00%	28.95%	71.05%
		Relative abundance	37.83%	56.52%	5.37%	0.04%	0.24%	0.00%	99.72%	0.28%

**Table 5:** Metagenomics-based relative abundance at the genus and species level.

Top Ten Genera Based on Relative Abundance				Top Ten Species Based on Relative Abundance			
Genus	DZ-FG_60d_	MC-FG_60d_	FC-FG_60d_	Species	DZ-FG_60d_	MC-FG_60d_	FC-FG_60d_
*Lactobacillus*	97.85%	33.93%	97.74%	*Lactobacillus acetotolerans*	96.20%	33.72%	96.08%
*Acetilactobacillus*	1.17%	65.54%	1.06%	*Acetilactobacillus jinshanensis*	1.18%	65.54%	1.06%
*Pichia*	0.24%	0.05%	0.32%	*Lactobacillus helveticus*	0.74%	0.04%	0.74%
*Saccharomyces*	0.12%	0.01%	0.22%	*Lactobacillus amylolyticus*	0.42%	0.02%	0.41%
*Lentilactobacillus*	0.13%	0.02%	0.13%	*Lactobacillus crispatus*	0.32%	0.12%	0.31%
*Acetobacter*	0.03%	0.17%	0.03%	*Pichia kudriavzevii*	0.24%	0.05%	0.32%
*Ligilactobacillus*	0.11%	0.02%	0.11%	*Saccharomyces cerevisiae*	0.12%	0.01%	0.22%
*Kroppenstedtia*	0.08%	0.04%	0.08%	*Ligilactobacillus acidipiscis*	0.11%	0.02%	0.11%
*Loigolactobacillus*	0.05%	0.03%	0.05%	*Lentilactobacillus buchneri*	0.11%	0.01%	0.11%
*Sphingomonas*	0.04%	0.02%	0.05%	*Lactobacillus amylovorus*	0.09%	0.01%	0.12%

## Data Availability

The original contributions presented in the study are included in the article, further inquiries can be directed to the corresponding author.
